# ASSOCIATION BETWEEN POSTURAL BALANCE AND ANTHROPOMETRIC INDEXES IN ELEMENTARY SCHOOLCHILDREN

**DOI:** 10.1590/1984-0462/;2018;36;1;00011

**Published:** 2017-11-13

**Authors:** Simone Lara, Susane Graup, Rodrigo de Souza Balk, Lilian Pinto Teixeira, Ariane Dias Farias, Giselle Baioco Alves, Verônica Benachio Leiria

**Affiliations:** a Universidade Federal do Pampa, Uruguaiana, RS, Brasil.

**Keywords:** Child, Postural balance, Nutritional status, Obesity, Overweight, Criança, Equilíbrio postural, Estado nutricional, Obesidade, Sobrepeso

## Abstract

**Objective:**

To analyze the association between postural balance and anthropometric indicators in elementary school students.

**Methods:**

This cross-sectional, descriptive and quantitative study included children enrolled in the first year of elementary school, of both sexes, in the age group of 6 to 7 years. Children with any physical or cognitive impairment, children who did not participate in all stages of the study evaluation, or those who failed to perform postural balance assessments were excluded. The children underwent a balance evaluation through a computerized dynamic posturograph, with sensory organization tests (TOSs) in six different sensory conditions. In order to verify the anthropometric indicators, the body weight and height measurements were evaluated for later calculation of the body mass index (BMI), which was categorized into four groups: low weight, normal, overweight and obesity.

**Results:**

Eighty children with a mean age of 6.2±0.8 years were included, being 47 girls (58.8%). The analysis of the anthropometric indicators identified that 26.3% of students were overweight and 15% were obese. The children had averages below the reference values considered for their age range in conditions III and VI. There was a negative association between condition V and BMI and a positive association between values below normal in condition VI with overweight and obesity.

**Conclusions:**

There were associations between excess body weight and values below normal in some balance conditions, indicating that the anthropometric indicators interfered in the children’s postural balance.

## RESUMO

**Objetivo:**

Analisar a associação entre o equilíbrio postural e indicadores antropométricos em escolares do primeiro ano do ensino fundamental.

**Métodos:**

Estudo transversal, descritivo e quantitativo que incluiu crianças regularmente matriculadas no primeiro ano do ensino fundamental, de ambos os sexos, na faixa etária de 6 a 7 anos. Foram excluídas as crianças com qualquer incapacidade física ou cognitiva, as que não participaram de todas as etapas de avaliação do estudo ou as que não conseguiram realizar a avaliação do equilíbrio postural. As crianças realizaram uma avaliação do equilíbrio por meio da posturografia dinâmica computadorizada (PDC), por meio dos testes de organização sensorial (TOSs) em seis diferentes condições sensoriais. Para verificar os indicadores antropométricos, foram avaliadas as medidas de peso corporal e estatura, para posterior cálculo do índice de massa corporal (IMC), sendo este categorizado em quatro grupos: baixo peso, normal, sobrepeso e obesidade.

**Resultados:**

Foram incluídas 80 crianças (6,2±0,8 anos), sendo 47 meninas (58,8%). A análise dos indicadores antropométricos identificou 26,3% de escolares com sobrepeso e 15% com obesidade. As crianças apresentaram médias abaixo dos valores de referência considerados para sua faixa etária nas condições III e VI. Foi verificada associação negativa entre a condição V com o IMC e uma associação positiva entre os valores abaixo da normalidade na condição VI com o sobrepeso e a obesidade.

**Conclusões:**

Foram verificadas associações entre o excesso de peso corporal e valores abaixo da normalidade em algumas condições do equilíbrio, indicando que os indicadores antropométricos interferiram no equilíbrio postural das crianças.

## INTRODUCTION

Through body movements, children interact and act dynamically in physical and social environments.^1^ However, for children to be able to act, they need body balance as a basic support.^2^ This is defined as a sensorimotor integration that guarantees the maintenance of posture.^3^ Postural control and stability are associated to the functions of the visual, proprioceptive and vestibular systems, as well as to neuromuscular control.^4^ During childhood, there is an improvement in the patterns of postural control for the performance of the activities of daily living, with the maturation of the proprioceptive function at around 3 or 4 years of age. The visual and vestibular systems appear to reach the adult level at the age of 15 or 16.^5^

In this context, in the first years of life, children are more dependent on visual information to the detriment of somatosensory and vestibular information, and it is only around 7 years of age that the information coming from these three sensorial channels is integrated in the same way as adults.^6^ According to Schimid et al.,^7^ there is a correlation between the development of balance control and age in the pediatric population, since there is a clear modification in the postural strategies between the ages of 7 and 11 years in children with typical development.

Considering childhood as the most important stage of growth and development, the assessment of postural balance in children is relevant in order to determine the factors related to possible balance disorders,^8^ since data report high rates of motor delays in Brazilian children.^9^ In fact, Alonso et al.^10^ reiterate that there are a number of factors which can compromise balance, including anthropometric ones.

In this sense, there seems to be a relationship between postural balance and nutritional status: overweight and obesity can influence a child’s motor skills and postural control capacity.^11^ Thus, larger size and body mass may contribute to postural instability in childhood.^4^ Although studies discuss the existence of different postural strategies and reduced balancing ability in overweight children,^12^ there is little research evidencing the influence of body mass on postural balance^13^, and the relationships between body balance and childhood obesity are little investigated.^3^

With regard to the instruments to evaluate the postural balance of individuals, computerized dynamic posturography (CDP) is a gold standard in the measurement of the motor and sensorial contribution in maintaining balance.^14^ CDP analyzes the visual, proprioceptive and vestibular information, their interaction with the Central Nervous System and the motor responses of lower limbs and body in general, through a platform with sensors that capture body movements in different situations.^15^ Although there is much data on postural stability in the pediatric population, reliable and complete information on postural balance in this age group is still lacking,^16^ justifying the evaluation of children through CDP.

Although studies have reported the influence of body weight on postural balance in children, the association between this variable and the neural systems responsible for postural stability is limited, which underlies the relevance of the present study. Thus, this study aimed to identify the association between postural balance and anthropometric indicators in schoolchildren from the first year of elementary school.

## METHOD

This is a cross-sectional, descriptive and quantitative study, which included a convenience sample of students from a public school in the city of Uruguaiana, in the countryside of the Brazilian State of Rio Grande do Sul, in the first half of 2015. The inclusion criteria were: being enrolled regularly in the first year of elementary school; participants of both genders; aged between 6 and 7 years of age. The exclusion criteria were: children with any physical or cognitive impairment attested by a medical report; who did not participate in all stages of the study evaluation; or who were unable to perform postural balance assessments.

It should be noted that all ethical principles were respected, in accordance with the Declaration of Helsinki (2008) and Resolution 466/12 of the Brazilian National Health Council, in which a meeting was held with the legal representatives of each student for the signature of the Informed Consent (EHIC). The project was approved by the Ethics Committee of *Universidade Federal do Pampa*, under Protocol No. 457.088.

**Figure 1:**
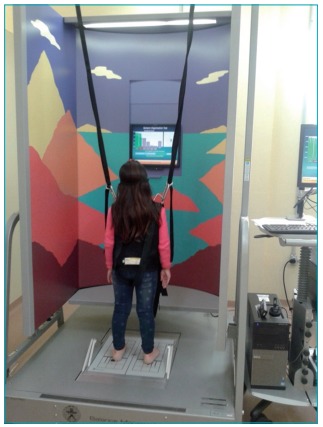
Postural balance evaluation through computerized dynamic posturography.

The children performed a postural balance assessment (Figure 1) through CDP (EquiTest^®^ System - NeuroCom International, Inc.). The equipment has a reference surface, in which the subject remains in the orthostatic position. On this platform, there are pressure sensors, activated by the displacement of the weight of the individual on the sole of the foot, in response to the displacement of the body. The reference surface is surrounded by a mobile visual field, which undergoes anteroposterior displacements.^17^

The assessment followed the criteria established by the manufacturer, including sensory organization tests (SOTs), and was conducted by a trained evaluator. For the examination, the child remained in an orthostatic and barefoot position, attached to the appliance by a vest, with their upper limbs hanging along their body and feet in a pre-designated location.

The SOTs present six conditions, which subject the individual to different sensory information, forcing them to use different strategies to maintain body balance. These tests consist of a noninvasive procedure that provides information on the integration and proportion of the visual, proprioceptive and vestibular components of balance^18^, as well as determine the performance of postural balance in the six different sensory conditions, evaluating the visual, proprioceptive and vestibular (SOT I, III and IV), proprioceptive and vestibular (SOT II and V), and proprioceptive (TOS IV) systems. The assessment of each condition lasts for 20 seconds and is repeated three times. The score varies from 0 to 100, and the higher the score, the better the postural balance of the subject. At the end of the test, the result is expressed by the total equilibrium index (composite) and by the sensory conditions evaluated. Normative data for six-year-old children were proposed in percentage by Casselbrant et al.^19^ and used as reference values in the present study, being: SOT I = 85%; SOT II = 80%; SOT III = 78%; SOT IV = 62%; SOT V = 43%; SOT VI = 46%; and composite score = 64%.

The following anthropometric indicators were evaluated: weight, measured in an anthropometric scale, with the children in light clothing and barefoot; stature, verified by means of a wall-mounted stadiometer, in an upright position, with the children barefoot; and body mass index (BMI), calculated by dividing body mass (kg) by the square of height (m). For the classification of BMI, the criteria of the Brazilian Sports Project (PROESP-Br) were used,^20^ which categorizes the results into four groups - low weight, normal weight, overweight and obesity.

For the data analysis, descriptive statistics were used primarily with the mean, standard deviation and frequency analysis values. To evaluate the normality of the data, the Kolmogorov-Smirnov test, which indicated a normal distribution, was applied. Differences in the SOT scores between the sexes were analyzed by Student’s *t*-test for independent samples. Analysis of the differences in frequency distributions between the sexes was performed by Fisher’s exact test. The correlation between SOTs and anthropometric variables was verified by the Pearson correlation test. The bivariate analysis was performed using the χ^2^ test, in which anthropometric indicators related to body mass (normal, overweight, obesity) were associated with dichotomized SOTs (normal and deficit). For all analyzes, p-value <0.05 was considered significant.

## RESULTS

Of a total of 119 children initially selected for the study, 39 were excluded because they did not meet the inclusion criteria established by the study. Thus, 80 children with a mean age of 6.2±0.8 years old were evaluated, with 47 of them being girls (58.8%). The analysis of the anthropometric indicators of the schoolchildren identified that 41.7% of them were out of normality patterns: 26.3% were overweight and 15% were obese. Considering genders, the female group presented overweight values of 31.9% and obesity values of 10.6%, while in the male group, the values were 18.2 and 21.2%, respectively.

The anthropometric characteristics of the sample are shown in Table 1, with no significant differences between the genders. In the same table, the postural balance profile of the children is presented by means of descriptive measures of the SOT, both for the general group and by gender, as well as the reference values for this sample. It was evidenced, in the general group, that the means of conditions III, VI and the composite score were below the reference values. Regarding gender, there was a better balance in girls when compared to the boys, in the condition I of the SOT (p=0.03).

**Table 1:** Descriptive values of the anthropometric characteristics and sensory organization tests of children.

Variable	Reference		General		Male		Female		p-value
	*X¯*	SD	*X¯*	SD	*X¯*	SD	*X¯*	SD	
Body mass (kg)	-	-	23.6	5.18	24.6	6.48	22.8	3.95	0.162
Stature (m)	-	-	1.15	0.07	1.16	0.07	1.15	0.07	0.592
BMI (kg/m^2^)	-	-	17.4	2.83	18.0	3.71	16.91	1.93	0.132
SOT I (%)	85	6	86.6	6.83	84.4	8.86	88.08	4.48	0.034*
SOT II (%)	80	8	83.8	6.68	83.4	7.39	84.20	6.19	0.591
SOT III (%)	78	8	77.9	11.8	77.7	9.97	77.99	13.09	0.923
SOT IV (%)	62	14	63.4	14.8	64.1	13.73	62.85	15.67	0.703
SOT V (%)	43	15	46.3	15.12	44.5	15.12	47.53	15.16	0.382
SOT VI (%)	46	16	40.8	19.16	38.2	18.89	42.44	19.39	0.351
Composite score (%)	64	8	60.9	11.28	60.0	10.77	61.51	11.70	0.553

*X¯*: mean; SD: standard deviation; BMI: body mass index; p-value: significant difference between the genders; SOT: sensory organization tests; *significant value; reference values: Casselbrant et al.^19^.

**Figure 2:**
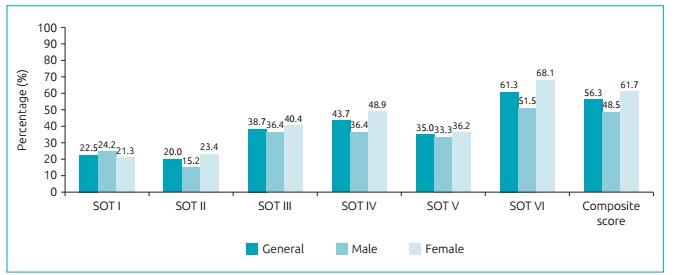
Frequency of schoolchildren with reference values below normal in sensory organization tests.

Figure 2 shows the frequency of schoolchildren with below-normal reference values in the SOTs. The majority of the individuals evaluated presented balance indicators below normal in SOT VI and in the composite score for the age, and these values were more expressive in the female group in comparison with the male group, but without statistical significance between the genders.

The correlation between the anthropometric variables and the SOT values is presented in Table 2, which shows an inversely proportional and significant association of the BMI with the SOT in condition V, in the general group and in the female gender.

**Table 2:** Correlation values between the anthropometric variables and the sensory organization test of the general group and according to gender.

Tests		General			Male			Female		
		BM	STA	BMI	BM	STA	BMI	BM	STA	BMI
SOT I	r	-0.020	-0.055	-0.133	0.062	0.071	-0.036	-0.045	-0.122	-0.189
	p	0.863	0.626	0.241	0.732	0.695	0.844	0.765	0.415	0.203
SOT II	r	-0.151	-0.130	-0.045	-0.215	-0.236	-0.045	-0.066	-0.048	-0.045
	p	0.183	0.250	0.692	0.229	0.186	0.803	0.660	0.746	0.763
SOT III	r	-0.010	-0.020	-0.121	0.145	0.033	-0.148	-0.083	-0.049	-0.094
	p	0.931	0.863	0.283	0.420	0.855	0.412	0.580	0.744	0.528
SOT IV	r	-0.098	-0.137	-0.103	-0.186	-0.190	-0.017	-0.049	-0.100	-0.152
	p	0.386	0.226	0.366	0.299	0.290	0.925	0.741	0.506	0.309
SOT V	r	-0.127	-0.209	-0.330	0.031	-0.032	-0.208	-0.225	-0.309	-0.392
	p	0.263	0.063	0.003	0.864	0.859	0.246	0.128	0.035	0.006
SOT VI	r	-0.091	-0.205	-0.168	-0.114	-0.162	-0.033	-0.052	-0.210	-0.248
	p	0.424	0.068	0.136	0.528	0.367	0.857	0.730	0.156	0.093
Composite score	r	-0.102	-0.161	-0.178	-0.101	-0.154	-0.113	-0.085	-0.149	-0.215
	p	0.369	0.153	0.115	0.576	0.391	0.532	0.568	0.317	0.148

r: correlation value; p: significance level; BM: body mass; STA: stature; BMI: body mass index; SOT: sensory organization test.

Table 3 shows the association between the values below normal in condition VI of the SOT with overweight and obesity in the general group. With regard to sex, it was possible to identify an association between overweight and obesity and values below normal in condition V of SOT in the female group.

**Table 3:** Association between values below normality (deficit) in sensory organization tests and body mass index in schoolchildren.

Tests		General		Male		Female	
		Overweight	Obese	Overweight	Obese	Overweight	Obese
SOT I	Deficit n (%)	6 (33.3)	3 (16.7)	1 (12.5)	2 (25.0)	5 (50.0)	1 (10.0)
	p-value	0.679		0.876		0.386	
SOT II	Deficit n (%)	6 (37.5)	3 (18.8)	1 (20.0)	2 (40.0)	5 (45.5)	1 (9.1)
	p-value	0.391		0. 496		0.556	
SOT III	Deficit n (%)	9 (29.0)	6 (19.4)	1 (8.3)	4 (33.3)	8 (42.1)	2 (10.5)
	p-value	0.541		0.312		0.451	
SOT IV	Deficit n (%)	11 (31.4)	7 (20.0)	1 (8.3)	4 (33.3)	10 (43.5)	3 (13.0)
	p-value	0.250		0.312		0.160	
SOT V	Deficit n (%)	10 (35.7)	6 (21.4)	2 (18.2)	3 (27.3)	8 (47.1)	3 (17.6)
	p-value	0.250		0.825		0.047*	
SOT VI	Deficit n (%)	16 (32.7)	9 (18.4)	3 (17.6)	5 (29.4)	13 (40.6)	4 (12.5)
	p-value	0.049*		0.483		0.083	
Composite score	Deficit n (%)	13 (28.9)	8 (17.8)	2 (12.5)	4 (25.0)	11 (37.9)	4 (13.9)
	p-value	0.518		0.677		0.262	

p-value: significance in the χ^2^ test; SOT: sensory organization test.

## DISCUSSION

In the present study, an expressive percentage of children with anthropometric indicators outside of the normal range was found. In relation to postural balance, the children presented values below the reference values considered in two of the six sensorial conditions evaluated. Regarding gender, a better balance was observed in girls when compared to boys in condition I of SOT. Regarding the associations between the variables of the study, the BMI showed an inversely proportional association with the balance, and there was an association between balance deficit and overweight and obesity in the children evaluated.

Regarding the anthropometric indicators, the present study showed a prevalence of 41.3% of children with above-adequate body weight, including 26.3% being overweight and 15% being obese. Likewise, expressive percentages of overweight and childhood obesity were also found in the study by Matsudo et al.,^21^ in which 485 children (aged 9 to 11 years old) were evaluated, with a prevalence of 23.1% overweight and 22.3% of obesity. Corroborating the data presented in this study, Ferreira et al.^22^ found a prevalence of 19.1% of overweight and 14.1% of obesity in 199 children aged 8 to 10 years old.

In the present study, girls had a significantly higher SOT condition I than boys. These findings are in line with the study by Alves et al.,^23^ who, through SOTs, compared the postural balance of 282 children regarding sex and found that girls, aged 8 years, presented higher values in relation to boys in condition I of SOT, indicating a slight anticipation of the beginning of their maturation period. Authors report that, as in other systems, the maturation of systems responsible for postural balance seems to develop earlier in girls than in boys,^24^ which could explain the differences found between genders in the present study.

In another study conducted with children aged between 7 and 10 years old, in which motor skills were compared between genders, the balance was higher in girls.^25^ In fact, Ruiz et al.^26^ report that static and dynamic balance skills tend to be better in girls than in boys.

In this study, there was a significant association of reference values below normality in condition VI of SOT with overweight and obesity in schoolchildren, while in the female group, this association occurred in condition V of SOT. This relationship has been widely described in the literature, with special emphasis on its repercussions on the development of the neuromotor system, as demonstrated by Gentier et al.^27^ These authors identified that obese children find it more difficult to integrate sensory information, which corroborates the findings of the present study, in which the nutritional condition negatively influenced the outcome of visual, vestibular and proprioceptive systems (condition VI of SOT).

Thus, Pau et al.^12^ suggest the existence of different postural strategies and reduced balancing ability among overweight children. From the neurophysiological point of view, the stretching of the skin in obese children is believed to increase the distance between the cutaneous mechanoceptors and to reduce the discrimination of somatosensory perception.^28^ Furthermore, the authors hypothesize that the body schema is constructed based on multisensory inputs, including cutaneous and proprioceptive receptors, and that, with obesity, these receptors can provide altered information of the somatosensory cortical area, modifying the representation of the body schema in obese individuals. In this perspective, there is evidence that some sensory receptors in obese individuals could be associated with postural instability.^29^ In addition, obesity causes a higher-than-expected pressure on children’s feet. This seems to be related to the decline in plantar sensitivity, leading to a smaller capacity to receive sensory information and to promote postural adjustments.^30^

Obese children experience greater difficulty in performing daily tasks due to poor postural stability, as shown by Lemos et al.^3^ In their review, these authors found that the difficulty in maintaining the body balance in obese children is mainly related to the physical modifications of the body, combined to the smaller amount of body experiences.

Regarding gender, in the present study, there was an association between excess body weight and reference values considered in the proprioceptive and vestibular systems (condition V of SOT) only in girls. Thus, it is believed that girls’ body balance is more affected by excess weight than boys’. Although studies have found that girls have a greater ability to use sensory information to maintain posture than boys,^23^ the study found that excess body weight seemed to affect that ability in girls.

Considering that the study subjects are in the development phase and are in an age group where consistent changes occur in postural stability, the fact that the collection was performed in just one moment can be considered as an important limitation of the study. However, the results provide considerable evidence that overweight is capable of negatively influencing the postural stability of children, which serves as a warning of a further consequence of obesity for child development. In addition, other limitations of the study that can be highlighted are the fact that the sample was selected by means of convenience, as well as the absence of a calculation of the sample size.

In conclusion, it can be said that, in the present study, associations between excess body weight and values below normal were observed in some balance conditions, suggesting that anthropometric indicators may interfere in the children’s postural balance.

## References

[1] Manoel E, Krebs RJ (2001). Criança e desenvolvimento: algumas notas numa perspectiva etária. Desenvolvimento Infantil em Contexto.

[2] Westcott SL, Burtner P (2004). Postural control in children: implications for pediatric practice. Phys Occup Ther Pediatr.

[3] Lemos LF, David AC, Teixeira CS, Mota CB (2009). Childhood obesity and its effect on corporeal balance. Acta Fisiatr.

[4] Mignardot JB, Olivier I, Promayon E, Nougier V (2013). Origins of balance disorders during a daily living movement in obese: can biomechanical factors explain everything?. PLoS One.

[5] Steindl R, Kunz K, Schrott-Fischer A, Scholtz AW (2006). Effect of age and sex on maturation of sensory systems and balance control. Dev Med Child Neurol.

[6] Bortolaia AP, Barela AM, Barela JA (2003). Postural control in 3 to 11 year-old children with visual impairment. Motriz.

[7] Schimid M, Conforto S, Lopez L, Renzi P, D'Alessio T (2005). The development of postural strategies in children: a factorial design study. J Neuroeng Rehabil.

[8] Moraes AG, David AC, Castro OG, Marques BL, Carolino MS, Maia EM (2014). Comparison of a single leg stance balance between children and adults. Rev Bras Educ Fís Esporte.

[9] Valentini NC, Coutinho MT, Pansera SM, Santos VA, Vieira JL, Ramalho MH (2012). Prevalence of motor deficits and developmental coordination disorders in children from South Brazil. Rev Paul Pediatr.

[10] Alonso AC, Vieira PR, Macedo OG, Greve JM (2007). Avaliação e reeducação proprioceptiva. Tratado Medicina de Reabilitação.

[11] Shultz SP, Byrne NM, Hills AP (2014). Musculoskeletal function and obesity: implications for physical activity. Curr Obes Rep.

[12] Pau M, Kim S, Nussbaum MA (2012). Does load carriage differentially alter postural sway in overweight vs. normal-weight schoolchildren?. Gait Posture.

[13] Alonso AC, Mochizuki L, Monteiro CB, Santos S, Luna NM, Brech GC (2012). Fatores antropométricos que interferem no equilíbrio postural. BJB.

[14] Mancini M, Horak FB (2010). The relevance of clinical balance assessment tools to differentiate balance deficits. Eur J Phys Rehabil Med.

[15] Quitschal RM, Fukunaga JY, Ganança MM, Caovilla HH (2014). Evaluation of postural control in unilateral vestibular hypofunction. Braz J Otorhinolaryngol.

[16] Barozzi S, Socci M, Soi D, Di Berardino F, Fabio G, Forti S (2014). Reliability of postural control measures in children and young adolescents. Eur Arch Otorhinolaryngol.

[17] Oda DT, Ganança CF (2015). Computerized dynamic posturography in the assessment of body balance in individuals with vestibular dysfunction. Audiol Commun Res.

[18] Hu M, Chen T, Dong H, Wang W, Xu K, Lin P (2015). Clinical values of the sensory organization test in vestibular diseases. Zhonghua Er Bi Yan Hou Tou Jing Wai Ke Za Zhi.

[19] Casselbrant ML, Mandel EM, Sparto PJ, Perera S, Redfern MS, Fall PA (2010). Longitudinal posturography and rotational testing in children three to nine years of age: Normative data. Otolaryngol Head Neck Surg.

[20] Gaya A, Lemos A, Gaya A, Teixeira D, Pinheiro E, Moreira R (2012). Projeto esporte Brasil: Manual de testes e avaliação.

[21] Matsudo VK, Ferrari GL, Araújo TL, Oliveira LC, Mire E, Barreira TV (2016). Socioeconomic status indicators, physical activity, and overweight/obesity in Brazilian children. Rev Paul Pediatr.

[22] Ferreira SD, Carballo FP, Sousa FF, Silva DM (2015). Prevalence and factors associated with overweight/obesity and systemic arterial hypertension in the private education network children of Divinópolis, MG. Cad Saúde Colet.

[23] Alves RF, Rossi AG, Pranke GI, Lemos LF (2013). Influence of gender in postural balance of school age children. Rev CEFAC.

[24] Cumberworth VL, Patel NN, Rogers W, Kenyon GS (2007). The maturation of balance in children. J Laryngol Otol.

[25] Miranda TB, Beltrame TS, Cardoso FL (2011). Motor performance and nutritional status of schoolchildren with and without developmental coordination disorder. Rev Bras Cineantropom Desempenho Hum.

[26] Ruiz LM, Graupera JL, Gutiérrez M, Miyahara M (2003). The assessment of motor coordination in children with the Movement ABC test: A comparative study among Japan, USA and Spain. Int J Appl Sport Sci.

[27] Gentier I, D'Hondt E, Shultz S, Deforche B, Augustijn M, Hoorne S (2013). Fine and gross motor skills differ between healthy weight and obese children. Res Dev Disabil.

[28] Mignardot JB, Olivier I, Promayon E, Nougier V (2010). Obesity Impact on the Attentional Cost for Controlling Posture. PLoS One.

[29] Wang L, Li JX, Xu DQ, Hong YL (2008). Proprioception of ankle and knee joints in obese boys and nonobese boys. Med Sci Monit.

[30] Rocha ES, Bratz DT, Gubert LC, David A, Carpes FP (2014). Obese children experience higher plantar pressure and lower foot sensitivity than non-obese. Clin Biomech (Bristol, Avon).

